# Use of Physiologically Based Kinetic Modeling-Facilitated Reverse Dosimetry to Predict *In Vivo* Acute Toxicity of Tetrodotoxin in Rodents

**DOI:** 10.1093/toxsci/kfac022

**Published:** 2022-02-26

**Authors:** Annelies Noorlander, Mengying Zhang, Bennard van Ravenzwaay, Ivonne M C M Rietjens

**Affiliations:** Division of Toxicology, Wageningen University, 6708 WE Wageningen, The Netherlands; Division of Toxicology, Wageningen University, 6708 WE Wageningen, The Netherlands; Division of Toxicology, Wageningen University, 6708 WE Wageningen, The Netherlands; Experimental Toxicology and Ecology, BASF SE, Ludwigshafen 67056, Germany; Division of Toxicology, Wageningen University, 6708 WE Wageningen, The Netherlands

**Keywords:** physiologically based kinetic modeling, reverse dosimetry, tetrodotoxin (TTX), neurotoxicity, new approach methodology

## Abstract

In this study, the ability of a new *in vitro*/*in silico* quantitative *in vitro*–*in vivo* extrapolation (QIVIVE) methodology was assessed to predict the *in vivo* neurotoxicity of tetrodotoxin (TTX) in rodents. *In vitro* concentration–response data of TTX obtained in a multielectrode array assay with primary rat neonatal cortical cells and in an effect study with mouse neuro-2a cells were quantitatively extrapolated into *in vivo* dose–response data, using newly developed physiologically based kinetic (PBK) models for TTX in rats and mice. Incorporating a kidney compartment accounting for active renal excretion in the PBK models proved to be essential for its performance. To evaluate the predictions, QIVIVE-derived dose–response data were compared with *in vivo* data on neurotoxicity in rats and mice upon oral and parenteral dosing. The results revealed that for both rats and mice the predicted dose–response data matched the data from available *in vivo* studies well. It is concluded that PBK modeling-based reserve dosimetry of *in vitro* TTX effect data can adequately predict the *in vivo* neurotoxicity of TTX in rodents, providing a novel proof-of-principle for this methodology.

Tetrodotoxin (TTX; Figure 1) is a naturally occurring neurotoxin that can be found in various marine gastropods and some fish species (Bane *et al.*, 2014; Centers for Disease Control and Prevention, 1996). There are over 30 structural analogs of TTX (Huang *et al.*, 2008). TTX has potent voltage-gated sodium channel blocker activity (Sui *et al.*, 2002), preventing depolarization, and propagation of action potentials in nerve cells, resulting in the loss of sensation (Bane *et al.*, 2014). The acute exposure to TTX leads to a wide range of acute adverse effects including skeletal muscle fasciculations, apathy, lethargy, ataxia, paralysis, and even death (Bane *et al.*, 2014).

**Figure 1.**
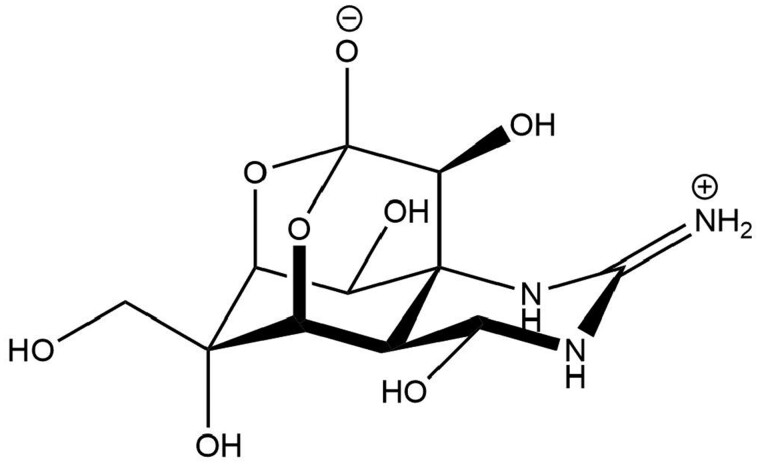
Structural formula of tetrodotoxin.

The European Food Safety Authority (EFSA) established an acute reference dose (ARfD) for TTX of 0.25 µg/kg bw based on an acute toxicity study with a single intragastric dose in mice with a no observed adverse effect level (NOAEL) of 75 µg/kg bw choosing apathy as the critical effect observed at a lowest observed adverse effect level (LOAEL) of 125 µg/kg bw (Abal *et al.*, 2017; EFSA Panel on Contaminants in the Food Chain [CONTAM] *et al.*, 2017). In this study, lethality was observed at 250 µg/kg bw with a steep dose–response curve from which a benchmark dose lower confidence limit (BMDL_10_) of 112 µg/kg bw could be derived (EFSA Panel on Contaminants in the Food Chain [CONTAM] *et al.*, 2017). Because this BMDL_10_ value for lethality was considered to be close to the NOAEL for apathy, the EFSA Panel argued that it cannot be excluded that effects can still occur at 75 µg/kg bw. Therefore, they established the ARfD based on the next lower test dose (25 µg/kg bw) using an uncertainty factor of 100 to derive the ARfD of 0.25 µg/kg bw. The EFSA opinion also provided an overview of median lethal dose (LD_50_) data from mouse studies upon different routes of exposure, indicating toxicity upon oral gavage or intragastric dosing, with LD_50_ values amounting to 232 µg/kg bw (Abal *et al.*, 2017) and 532 µg/kg bw (Xu *et al.*, 2003), to be substantially lower than the LD_50_ values reported upon intraperitoneal or subcutaneous dosing, for which LD_50_ values ranged from 9 to 12.5 µg/kg bw (Kao, 1966; Kao and Fuhrman, 1963; Marcil *et al.*, 2006; Xu *et al.*, 2003). In addition, the LD_50_ in rats upon intramuscular (IM) administration was reported to amount to 10–11.1 µg/kg bw (Hong *et al.*, 2017; Marcil *et al.*, 2006), whereas Finch *et al.*, (2018) and Hong *et al.*, (2018) reported LD_50_ values for rats upon oral dosing of 909 and 571.43 µg/kg bw, respectively, that were not included in the EFSA overview.

The available TTX data for human are too limited to provide a point of departure (PoD) for risk assessment, with only a minimum lethal oral dose of 2 mg being mentioned in literature, which is equivalent to 40 µg/kg bw for a 50 kg Japanese subject (EFSA Panel on Contaminants in the Food Chain [CONTAM] *et al.*, 2017). Additionally, Kasteel and Westerink (2017) proposed an ARfD of 1.33 µg/kg bw for human based on a so-called universal mammalian LD_50_ of 400 µg/kg bw derived from reported oral LD_50_ values in mice (334–700 µg/kg bw). They applied a conservative factor of 10 to go from an LD_50_ value to a LOAEL value (40 µg/kg bw) and added another factor of 3 to obtain a NOAEL value (13.3 µg/kg bw). Finally, they took a factor of 10 into account for intraspecies differences.

Given the available data sets on acute toxicity of TTX in rodents and the many analogs of TTX for which experimental toxicity data are lacking, it is of interest to study whether the acute toxicity of TTX can be adequately predicted by a new approach methodology (NAM) such as quantitative *in vitro–in vivo* extrapolation (QIVIVE) using physiologically based kinetic (PBK) modeling with integrated *in vitro* and *in silico* data and applying reverse-based dosimetry. Thus, this study aimed to evaluate the potential of using *in vitro* toxicity data obtained with primary rat neonatal cortical cells on a multielectrode array (MEA) assay or an effect study in mouse neuro-2a cells combined with PBK model-based reverse dosimetry to predict the *in vivo* acute neurotoxicity of TTX in rodents. As TTX is hardly metabolized, highly hydrophilic, and has been identified as a substrate for organic cation transporters in the kidneys (Matsumoto *et al.*, 2017), active renal transport can be expected to contribute substantially to the *in vivo* TTX kinetics and has to be accounted for in the PBK models to be developed to facilitate the QIVIVE.

## MATERIALS AND METHODS

### Materials

TTX ≥98% (CAS 4368-28-9), was purchased from Sigma-Aldrich (Zwijndrecht, The Netherlands). Dimethyl sulfoxide (DMSO) was purchased from Acros Organics (Geel, Belgium) and phosphate-buffered saline was purchased from Invitrogen (Breda, The Netherlands). Pooled hepatocytes from male Sprague Dawley rats, cryopreserved hepatocyte recovery medium (CHRM, CM7000), and primary hepatocytes thawing and plating supplements (CM4000) were purchased from Thermo Fisher (Landsmeer, The Netherlands).

### Methods

#### Clearance of TTX

It was assumed that the *in vivo* acute toxicity of TTX is induced by the parent compound as there is no evidence of potential metabolites exerting a similar effect. Therefore, only the overall hepatic clearance of TTX was included in the PBK model. Primary hepatocytes from male Sprague Dawley rats were used to determine the hepatic clearance by the substrate depletion approach. To this end, pooled primary hepatocytes were thawed in a 37°C water bath and transferred to 50 ml CHRM (CM7000). The cell suspension was centrifuged at 100*g* for 15 min at room temperature, and the supernatant was removed. The collected hepatocytes were dissolved in 1 ml prewarmed hepatocyte incubation medium, which contained 4% primary hepatocyte thawing and plating supplements (CM4000) in Williams’ Medium E1 without phenol red. The density and viability of the hepatocytes were measured using the Cellometer (Auto T4, Nexcelom Bioscience). Hepatocytes with >90% viability were used for the incubation. The cells were diluted with incubation medium to reach a density of 1 × 10^6^ cells/ml. TTX was dissolved in DMSO to obtain a stock solution of 600 µM. A total of 20 µl Stock solution of TTX was added to 1980 µl medium to generate the exposure medium (final DMSO concentration 1% v/v). The exposure medium was preincubated for 5 min. The incubation was started by adding 100 µl primary hepatocytes into 100 µl preincubated exposure medium, giving a final concentration of 0.5 × 10^6^ cells/ml and 3 µM TTX (a nontoxic concentration to hepatocytes as shown by the WST-1 assay [data not shown]) (final DMSO concentration 0.5% v/v). The incubation was done using a shaker (Titramax 1000, Heidolph, Germany) at 150 rpm in a 5% CO_2_, 95% air-humidified incubator. The incubation time points were: 0, 1, 2, 3, 4, 5, 7, 8.5, 10, 15, 30, 45, 60, and 90 min. For each incubation time point a corresponding control was included, consisting of an incubation performed in the absence of primary hepatocytes. The incubation was terminated by adding 100 µl cold acetonitrile and the samples were put on ice for 30 min, then centrifuged at 3500 rpm (1200 *g*) for 15 min at 4°C. Supernatants were collected and the concentration of TTX was quantified using liquid chromatography-mass spectrometry (LC–MS/MS) analysis. All incubations were performed in triplicate in 3 independent studies. The ratio of the remaining parent compound concentration in the incubation sample (*C*_compound_) and in the sample at time 0 as control (*C*_control_) was calculated for each incubation time (taking the amount of TTX left in the corresponding control incubations into account) and the depletion curve of the parent compound [ln (*C*_compound_/*C*_control_)] against time was derived. The slope of the linear part of the depletion curve represents the elimination rate constant (*k*, in 1/min) of the parent compound. The *in vitro* clearance (CL_int,_  _*in vitro*_) of the parent compound was calculated using the following equation: CL_int,_  _*in vitro*_ (ml/min/10^6^ cells) = *k* (1/min)/V (10^6^ cells/ml; Obach, 1999; Sjögren *et al.*, 2009). *V* represents the number of hepatocytes per milliliter incubation mixture, (0.5 × 10^6^ cells/ml). The *in vitro* CL_int_ of the parent compound was scaled to a whole liver using the scaling factor of 135 000 (cell density liver expressed in 10^6^ cells/kg; Houston, 1994).

#### Analysis of TTX by LC–MS/MS

LC*–*MS/MS analysis was performed on a Shimadzu Nexera XR LC-20AD SR ultra high performance liquid chromatograhy (UHPLC) system coupled with a Shimadzu LCMS-8045 mass spectrometer (Shimadzu Benelux’s Hertogenbosch, The Netherlands). The samples (1 µl) were loaded onto a BEH C18 column (1.7 µm, 2.1 × 100 mm) at a flow rate of 0.3 ml/min. The column temperature was set to 40°C. The mobile phase consisted of ultrapure water with 0.1% (v/v) formic acid as mobile phase A and acetonitrile containing 0.1% (v/v) formic acid as mobile phase B. The initial condition of the eluents was 5% A, then changed to 50% A in 2 min and subsequently returned to the initial condition in the next 5 min, and was kept at these starting conditions for another 5 min. The total runtime was 12 min. A Shimadzu LCMS-8045 triple quadrupole with electrospray ionization interface was used to perform the MS*–*MS analysis. The instrument was operated in the positive ion mode in the multiple reaction monitoring mode with a spray voltage of 4.5 kV. TTX was monitored at the [M + H]^+^ of precursor to product 320.1 > 302.19, 320.1 > 162.4, and 320.1 > 60.2 *m/z.* The Postrun Analysis function from the LabSolutions software (Shimadzu, Kyoto, Japan) was used to obtain the peak area of the total ion chromatogram.

#### Development of the PBK model for TTX

**Figure 2.**
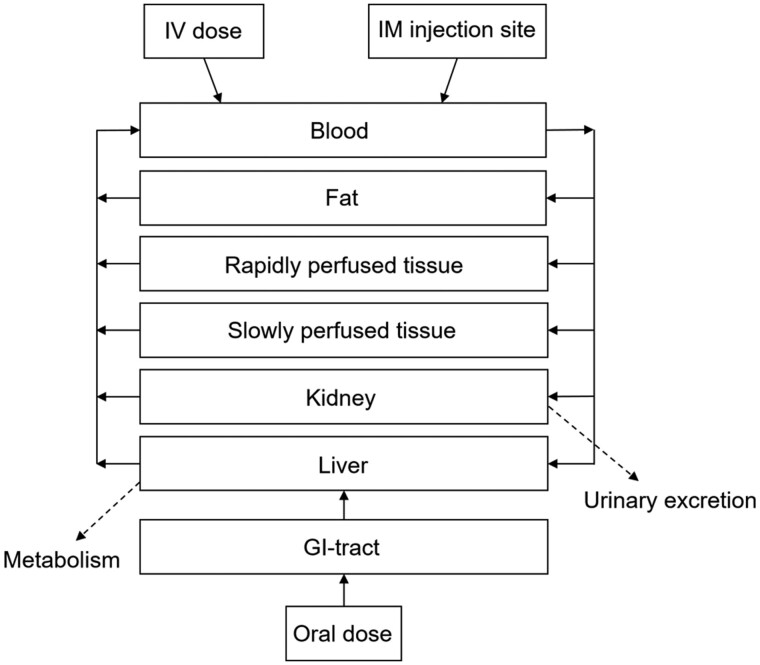
Schematic representation of the rodent physiologically based kinetic model for tetrodotoxin.

The generic PBK model developed in our previous study (Zhang *et al.*, 2020) was used with minor modifications, defining a PBK model for TTX in both rat and mouse. To allow model evaluation based on the 3 available *in vivo* kinetic data sets, all with different administration routes, the PBK model was built for oral, intravenous (IV), and IM administration (Hong *et al.*, 2017, 2018). For the IM administration, it was assumed that TTX was solely taken up in the blood via the muscle tissue at the injection site, thereby excluding the role of the subcutaneous and lymph routes. To distinguish between IM and IV administration, the rate of absorption from the IM injection site was set to a much lower value (50 h^−1^) than that for an IV injection (1 000 000 h^−1^). Given that the study by Hong *et al.* (2017) concluded that the predominant route of the elimination of TTX is by urinary excretion, a kidney compartment was included in the PBK models. The developed PBK model for TTX consisted of 8 compartments, including the site of injection, GI-tract, blood, fat, liver, kidney, rapidly perfused tissue, and slowly perfused tissue. The schematic representation of the PBK model is displayed in Figure 2. A separate compartment for the brain was not included given the inability of TTX to pass the blood–brain barrier (Melnikova *et al.*, 2018). The values for physiological and anatomical parameters for rat were obtained from Brown *et al.* (1997b) and for mouse from Hall *et al.* (2012). The partition coefficients to describe the distribution of TTX over the different tissues were estimated using the quantitative property-property relationship approach from Rodgers and Rowland (2006) facilitated by a QIVIVE toolbox (input: zwitterion, pKa1: 8.76, pKa2: 11 logP: −6.2 and molecular weight: 319.27 g/mol; Punt *et al.*, 2020). The assumption was made that the distribution of TTX in rat is the same as in mouse. The glomerular filtration (GF) was added to the model according to the equation: GF = GFR × (CVK × fub_*in vivo*_), where GFR is the GF rate, which is 5.2 ml/min/kg bw for rat and 14 ml/min/kg bw for mouse (Walton *et al.*, 2004), CVK is the concentration of TTX in the kidney compartment and fub_*in vivo*_ is the fraction of TTX unbound in the *in vivo* situation. As mentioned by Matsumoto *et al.* (2017), TTX seems to be a substrate for some active transporters in the proximal tubule cells in the kidney. Since it is unknown which transporter has the highest contribution it was decided to work with an estimated apparent overall *V*_max_ and *K*_m_; 1 *V*_max_ and 1 *K*_m_ for all transporters involved. After running model predictions including only GF as the excretion pathway, data for the apparent overall *V*_max_ and *K*_m_ were estimated based on manual input of *V*_max_ and *K*_m_ searching for the optimal transporter efficiency (TE = *V*_max_/*K*_m_ in µl/min/mg protein) by fitting to the available *in vivo* kinetic data. Matsumoto *et al.* (2017) reported Papp values for the bidirectional transport of TTX over an LLC-PK1 kidney cell layer. Future use of such Papp values to define the *in vivo* kinetic parameters for urinary excretion of TTX in a PBK model requires definition of the scaling factor(s) needed to convert these *in vitro* Papp values to the kinetic constants for active transport of TTX in the kidney *in vivo*. This scaling could be achieved by scaling the model predictions to fit available *in vivo* data, as done in this study for *V*_max_, as well as in other studies for other transport parameters, including parameters for renal excretion in PBK models, such as for the PBK model for perfluorooctanoic acid in rats (Worley and Fisher, 2015) and the PBK model for mepiquat in rats (Noorlander *et al.*, 2021). The model equations were coded and numerically integrated in Berkeley Madonna 8.0.1 (UC Berkeley, California), using the Rosenbrock’s algorithm for stiff systems (see Supplementary Material A for the model codes).

#### PBK model evaluation

For evaluation of the PBK model, *in vivo* kinetic data of the TTX blood concentration in time upon oral, IV or IM dosing were available for rats (Hong *et al.*, 2017, 2018). It was assumed that evaluation of the model in rats would support its use for mice as well. It is important to note that the *in vivo* kinetic data were obtained in plasma, whereas the PBK model predicts the concentration in whole blood. Thus, an adjustment of the reported concentrations in plasma to concentrations in blood was made by multiplying the plasma data with the blood:plasma ratio (0.42; derived from Hong *et al.* 2017). For the IM administration kinetic data presented by Hong *et al.* (2017) for the dried plasma curve were used since these data were corrected for the formed tritiated water by the hydrogen–tritium exchange of 11-[^3^H]TTX in the plasma possibly interfering with the results. To identify the most influential parameters of the PBK model on the model prediction of the maximum blood concentration (*C*_max_) upon oral and IM administration, a sensitivity analysis was performed (see Supplementary Figure 1). To this end, an initial input parameter value was increased by 5% and the sensitivity coefficients (SCs) were calculated using the equation SC = (*C*′–*C*)/(*P*′–*P*) × (*P*/*C*), in which *P* and *P*′ represent the initial and modified parameter value, respectively, whereas *C* and *C*′ are the initial and modified model output for *C*_max_ (Evans and Andersen, 2000). Each parameter was analyzed individually by changing 1 parameter at a time keeping the other parameters at their original value, while the total blood flow fraction was kept as 1. The sensitivity analysis was performed for exposure to 6 µg/kg for the oral and IM routes, representing the dose level actually used in the available *in vivo* studies (Hong *et al.*, 2017, 2018).

#### Translation of the in vitro neurotoxicity data for TTX to in vivo dose– response data

For rat, 2 *in vitro* concentration–response data sets were available. Both studies performed the MEA assay using primary rat neonatal cortical cells for measuring neuronal activity upon exposure to TTX (Kasteel and Westerink, 2017; Nicolas *et al.*, 2014). For mouse, 4 *in vitro* concentration–response data sets were available, where in all studies mouse neuro-2a cells were used to detect the inhibition by TTX on cellular toxicity (Hamasaki *et al.*, 1996; Kogure *et al.*, 1988; Nicolas *et al.*, 2015; Yamashoji and Isshiki, 2001; Yeo *et al.*, 1996). The inhibition is induced by first exposing the cells to veratridine (sodium-channel opener) and ouabain (blocking of Na^+^/K^+^-ATPase) which may result in disturbance of the sodium ion homeostasis in the cells resulting in cell death (Kogure *et al.*, 1988; Rossini and Hartung, 2012). When the veratridine/ouabain-treated cells are exposed to TTX the sodium channels are blocked and sodium accumulation in the cells is prevented, counteracting the toxic effects of veratridine and ouabain, thereby leading to cell survival (Kogure *et al.*, 1988). Throughout this study, this assay is further referred to as the neuro-2a assay. The available concentration–response data were used to predict the dose levels that were required to reach the respective effect concentrations of TTX in blood, using PBK modeling-based reverse dosimetry. It is of importance to realize that only the free fraction of the compound will exert the effects, which implies that a correction for protein binding prior to applying reverse dosimetry should be considered. However, due to the physicochemical characteristics of TTX the fraction unbound *in vivo* is 1 (see toolbox Punt *et al.*, 2020) and therefore, given the excellent water solubility of TTX, it was assumed that in the *in vitro* MEA medium and neuro-2a medium containing 10% fetal bovine serum the fu_*in vitro*_ of TTX was 1, too. Thus, the *in vitro* effect concentration (EC_*in vitro*_) of TTX was set equal to an *in vivo* effect concentration (EC_*in vivo*_), without a need for correction for potential differences in protein binding in the *in vitro* and *in vivo* situation. The *C*_max_ was the chosen dose metric for reverse dosimetry of TTX as the mode of action of its toxicity, sodium channel blocking, shows to be a concentration-dependent endpoint with a threshold (Rietjens *et al.*, 2019). So, the estimated EC_*in vitro*_ was set equal to *C*_max_ of TTX in the PBK model. By repeating these steps for all the *in vitro* test concentrations, the *in vitro* concentration–response data were converted to define the corresponding *in vivo* dose–response data.

#### Comparing predicted dose–response curves to in vivo toxicity data

After evaluation of the model, the predicted *in vivo* dose–response curves were compared with the available *in vivo* TTX toxicity data. For this comparison, the most sensitive endpoint for toxicity was chosen for each route and species as described in the result section. To quantify the comparison benchmark dose (BMD) responses were generated using the EFSA online BMD software. The BMD_10_ and BMDL_10_ were determined under the EFSA default settings for Akaike information criterion being 2 and a confidence interval of 95%. Only BMD_10_ and BMDL_10_ values as a result of model averaging were taken. Furthermore, a medium effective dose (and concentration) (ED_50_ and EC_50_) was calculated in Excel using the TREND function as follows: (1) calculate from the dataset the halfway response: $\mathrm{lowest}\;y_{\mathrm{value}}+\;(\mathrm{highest}\;y_{\mathrm{value}}\;–\;\mathrm{lowest}\;y_{\mathrm{value}})/2$ and (2) use the TREND function, which includes the 2 *x*-values with corresponding *y*-values in between where the halfway response lies to calculate its *x*-value, also the ED_50_ (or EC_50_). EC_50_ values were used to compare the *in* vitro data sets.

#### Human model

In spite of the limited available human data on TTX kinetics, a human PBK-model was defined, assuming that the evaluation of the model in rat would support its use for humans. The physiological and physicochemical parameters for the human model were taken from the literature in a similar way as for rat (Brown *et al.*, 1997a; Punt *et al.*, 2020) and are presented in Table 1. The kinetic constants were taken from the rat model and adjusted to human using human scaling factors. For reverse dosimetry 1 *in vitro* data set was available (Kasteel and Westerink, 2017) describing a concentration–response curve for the effect of TTX on human-induced pluripotent stem cell (hIPSC)-derived iCell neurons in coculture with hIPSC-derived iCell astrocytes in the MEA assay. Using our human PBK model, this *in vitro* concentration–response curve was translated to an *in vivo* dose–response curve for oral exposure to TTX and a BMD_10_, BMDL_10_, and ED_50_ were derived that were compared with available (on mouse study based) human data on TTX toxicity.

**Table 1.** Physiological and Anatomical Parameter Values and the Partition Coefficients Used for the Physiologically Based Kinetic Models

Parameters	Rat^a^	Mouse^b^	Human^a^
Body weight (kg)	0.24	0.03	70
Fraction of tissue volumes			
Fat	0.070	0.070	0.214
Liver	0.034	0.055	0.026
Blood	0.074	0.067	0.079
Kidney	0.007	0.017	0.004
Rapidly perfused tissue	0.091	0.137	0.064
Slowly perfused tissue	0.724	0.654	0.613
Cardiac output	15^c^	15.4^d^	15^c^
Fraction of blood flow to tissue			
Fat	0.070	0.070	0.052
Liver	0.174	0.158	0.227
Kidney	0.141	0.114	0.175
Rapidly perfused tissue	0.093	0.516	0.195
Slowly perfused tissue	0.512	0.142	0.351
Partition coefficients^e^			
LogP_ow_	−6.2^f^		
pKa1	8.76^g^		
pKa2	11^g^		
Fat/blood partition coefficient	0.46	0.46	0.46
Liver/blood partition coefficient	4.29	4.29	4.29
Kidney/blood partition coefficient	4.70	4.70	4.70
Rapid perfused tissue/blood partition coefficient	4.29	4.29	4.29
Slowly perfused tissue/blood partition coefficient	0.95	0.95	0.95

a Brown *et al*. (1997a).

b Hall *et al.* (2012).

c l/h × kg × bw^0.74^.

d l/h × kg × bw^0.75^.

e Punt *et al*. (2020).

f Hort *et al.* (2020).

g Camougis *et al.* (1967).

## RESULTS

### Substrate Depletion of TTX

**Figure 3.**
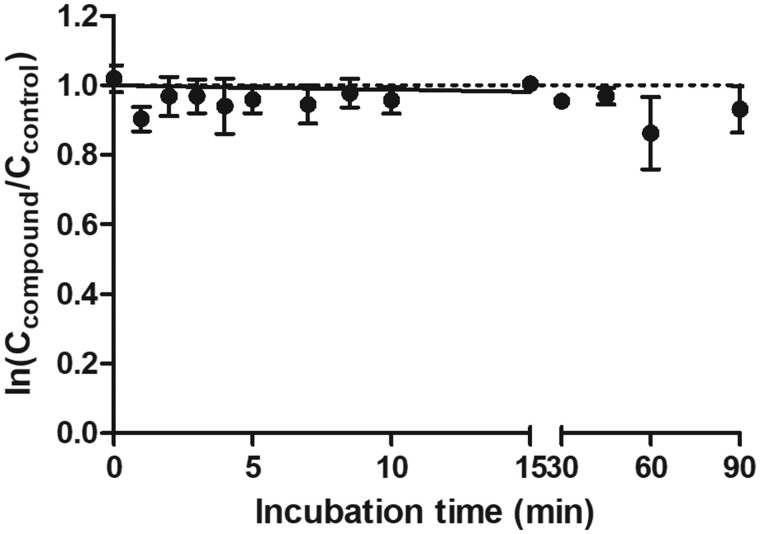
Time-dependent substrate depletion of tetrodotoxin in incubations with primary rat hepatocytes. Symbols represent the average ln (*C*_compound_/*C*_control_) at different incubation time points (mean ± SD of 3 independent experiments). Straight line represents the depletion curve and the dotted line represents zero depletion.

Figure 3 shows the depletion of TTX in incubations with rat hepatocytes. The *in vitro* hepatic clearance (CL_int_) derived from these data amounted to 1.6 × 10^−7^±0.01 ml/min/10^6^ cells converted to an *in vivo* CL_int_ of 1.1 × 10^−5^ l/h for the rat, indicating that clearance of TTX via metabolism is limited. It was assumed that the mouse hepatic clearance would be similarly limited.

#### *In Vitro* Concentration–Response Data for TTX in Rodent Cells

**Figure 4.**
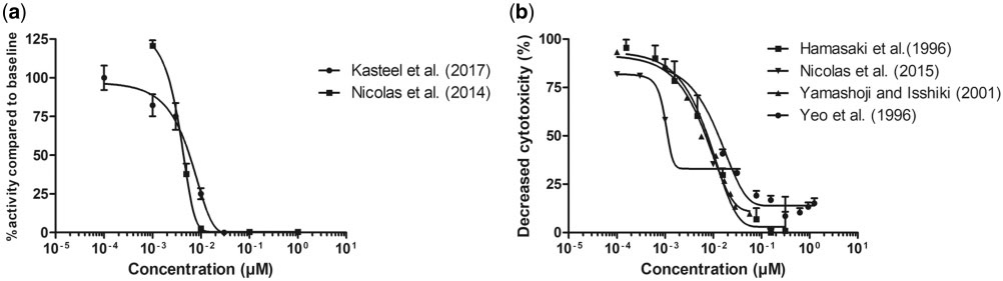
*In vitro* concentration–response curves for the effects of tetrodotoxin on (A) primary rat neonatal cortical cells in the MEA assay (circles: Kasteel and Westerink, 2017; EC_50_ = 0.0055 µM; squares: Nicolas *et al.*, 2014; EC_50_ = 0.0035 µM) and (B) neurotoxicity in mouse neuro-2a cells (squares: Hamasaki *et al.*, 1996; EC_50_ = 0.0075 µM; inverted triangles: Nicolas *et al.*, 2015; triangles: Yamashoji and Isshiki, 2001; EC_50_ = 0.0053 µM; and circles: Yeo *et al.*, 1996; EC_50_ = 0.0121 µM). Data points represent mean (± SD/SEM, where available).

The available *in vitro* concentration–response data for neurotoxicity of TTX in rat primary neonatal cortical cells in the MEA assay and for the neurotoxicity of TTX in the mouse neuro-2a assay are summarized in Figure 4. Both the rat MEA data (Figure 4A) and the mouse neuro-2a data (Figure 4B) reported in different studies provide comparable results, except for the data of Nicolas *et al.*, (2015) for the mouse neuro-2a assay, which indicate at a somewhat greater sensitivity. Nevertheless, these data together provide a suitable data set for QIVIVE and conversion into *in vivo* dose–response curves. Given the similarity of the concentration–response curves for the TTX-induced neurotoxicity in the neuro-2a assay reported in the studies of Hamasaki *et al.* (1996), Yamashoji and Isshiki (2001), and Yeo *et al.* (1996) these 3 data sets were used for the mice predictions by reverse dosimetry. The graphs in Figure 4 show that the rat primary neonatal cortical cells in the MEA assay (EC_50_ values 0.0035 and 0.0055 µM; Figure 4A) seem to be only slightly more sensitive than the mouse neuro-2a cells in the neuro-2a assay (EC_50_ values of the 3 corresponding data sets amounting to 0.0082 µM).

### PBK Model Evaluation

**Figure 5.**
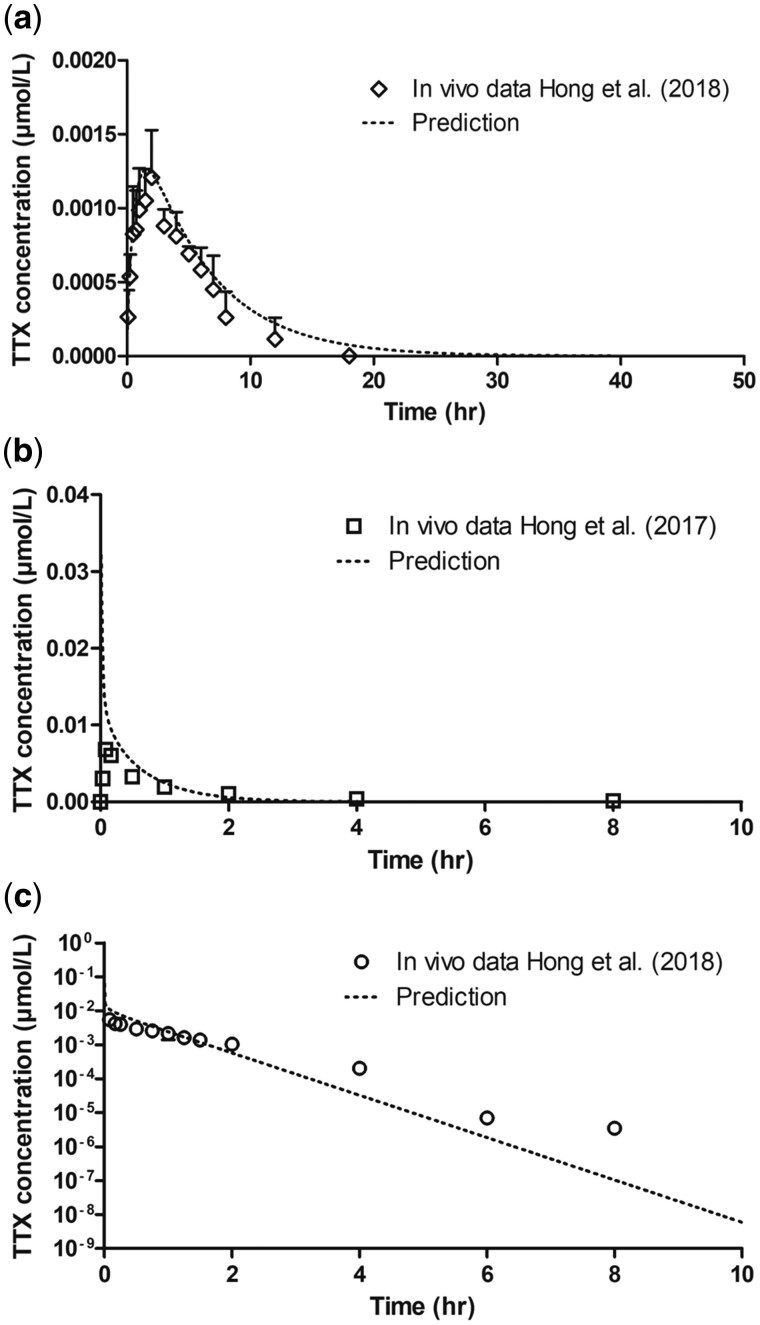
Predicted concentration time curves of tetrodotoxin (TTX) in whole blood of rat (striped lines) dosed with TTX via (A) oral (diamonds), (B) intramuscular (IM; squares), and (C) intravenous (IV; circles) administration. The literature data reported as plasma concentrations were adjusted to blood concentrations assuming a blood:plasma ratio of 0.42 (Hong *et al.*, 2017). Dosage used: oral 100 µg/kg bw with 6.7% bioavailability, IM and IV 6 µg/kg bw. Data points represent mean (± SD/SEM, where available).

The evaluation of the PBK model using different routes of administration and the parameter input presented in Table 1 is shown in Figure 5. Predictions were fitted to the *in vivo* data by estimating the rate of absorption for the oral route (ka: 0.18/h) and IM route (kb: 50/h) and optimizing the contribution of active renal excretion based on the transporter efficiency, which was 90 µl/min/mg protein (*V*_max_ = 180 pmol/min/mg protein, *K*_m_ = 2 µM). It appeared important to include this active excretion since it accounts for a substantial improvement in the predictions (compare Figure 5 with renal excretion, to Supplementary Figure 2 for data without taking renal excretion into account). For all administration routes, oral (Figure 5A), IM (Figure 5B), and IV (Figure 5C), the model was able to adequately predict the *in vivo* data. To enable subsequent PBK model-based reverse dosimetry for both the oral and the IM mode of administration a plot of the dose against *C*_max_ was made, which was used to convert the *in vitro* concentrations from Figure 4 to *in vivo* dose levels to generate the dose–response curves. As explained in the Materials and Methods section, the high water solubility of TTX eliminated the need for a correction for differences in protein binding with the fub *in vitro* and *in vivo* both being 1.

### Literature Reported *In Vivo* Dose–Response Data in Rodents

**Figure 6.**
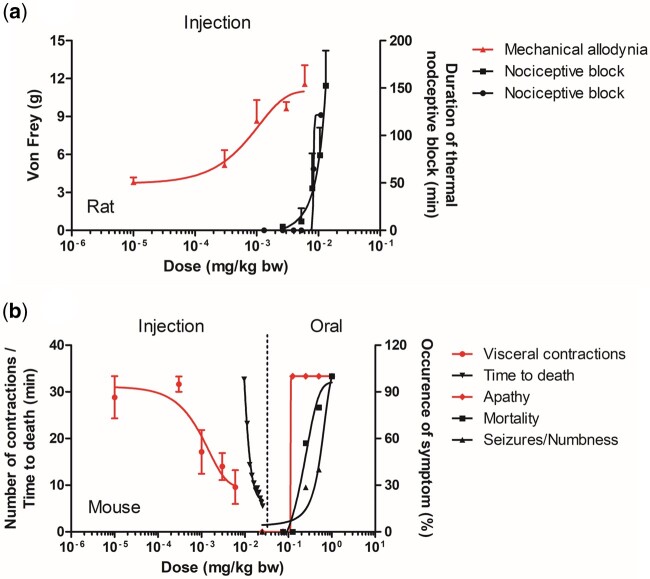
Overview of *in vivo* dose–response data for tetrodotoxin (TTX) in rodents found in literature including (A) *in vivo* data sets for rat after TTX injection: triangles; Von Frey (g) hair test; (Marcil *et al.*, 2006; left *y*-axis), squares and circles; duration of the nociceptive block (min; Kohane *et al.*, 1998; right *y*-axis, and Kohane *et al.*, 2000, respectively) and (B) *in vivo* data sets for mouse: either upon injection: circles; writhing test (Marcil *et al.*, 2006), inverted triangles; time to death (min; Finch *et al.*, 2018; left *y*-axis) or after oral administration: diamonds; apathy (%; Abal *et al.*, 2017), squares; mortality (%; Abal *et al.*, 2017), triangles; numbness and seizures (%; Abal *et al.*, 2017; right *y*-axis). The red data sets present the dose–response curves for the most sensitive endpoint that were chosen for evaluation of the QIVIVE predictions. Data points represent mean (± SD/SEM, where available).

Figure 6 summarizes the available *in vivo* dose–response data for TTX in rodents available in literature for evaluation of the QIVIVE predictions. It must be noted that although the reported *in vivo* data are used for evaluation of the QIVIVE predictions made in this study using a NAM, this does not imply that the authors of this study agree with the ethics of these animal studies as they involve pain and discomfort to the animals. Three data sets for rat (Figure 6A) originate from studies reporting on the pharmacological application of TTX as a morphine-like painkiller (Kohane *et al.*, 1998, 2000; Marcil *et al.*, 2006). The dose–response curves from these 3 studies reveal substantial differences in sensitivity depending on the endpoint used to quantify the effect. The data reported by Marcil *et al.* (2006) using the so-called Von Fray hair test to quantify the TTX-induced reduction in mechanical allodynia (pain) showed effects at 16-fold lower dose levels (Figure 6A, left *y*-axis) than the dose–response curves defined based on TTX-induced thermal nociceptive blocking (blocking of the peripheral sensory neurons; nociceptors; Figure 6A, right *y*-axis). The route of administration in all 3 studies was comparable consisting of subcutaneous/percutaneous injection. The data set reporting mechanical allodynia, apparently relating to the most sensitive endpoint, was selected for QIVIVE-based predictions. For mouse (Figure 6B), 6 data sets were identified in the available literature of which 2 related to parenteral administration and 4 to oral administration (Abal *et al.*, 2017; Finch *et al.*, 2018; Marcil *et al.*, 2006). Here too, the data sets for the parenteral route differ markedly, as the endpoint “time to death” in minutes requires higher doses to be affected than the more sensitive endpoint including a so-called writhing test where the number of contractions of the abdomen was measured after exposure to acetic acid following increasing concentrations of TTX (both shown on left *y*-axis of Figure 6B). The latter study was selected for further QIVIVE-based predictions. The data sets for the oral route show a lower sensitivity to TTX compared with the parenteral route likely related to the low oral bioavailability of TTX of 6.7% reported by Hong *et al.* (2018). For the oral route, dose–response curves for the macroscopically observed neurological symptoms apathy, numbness, seizures, and mortality were available (shown on right *y*-axis of Figure 6B), where apathy was the most sensitive endpoint and therefore selected for the QIVIVE-based predictions.

### QIVIVE to Translate *In Vitro* Neurotoxicity Data for TTX into *In Vivo* Dose–Response Data

**Figure 7.**
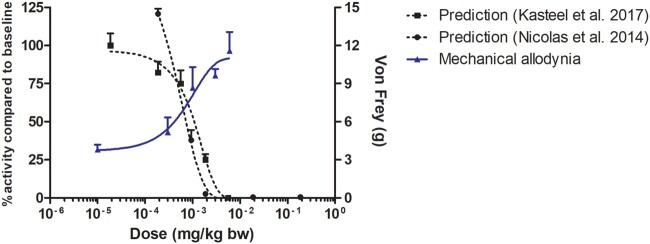
Predicted *in vivo* dose–response curve for tetrodotoxin in rat upon injection (intramuscular model) on the left *y*-axis compared with the *in vivo* data reported by Marcil *et al.* (2006) in the Von Frey hair test (blue line and triangles) on the right *y*-axis. The predictions were based on the rat multielectrode array assay data reported by Nicolas *et al.* (2014; black circles) or Kasteel and Westerink (2017; black squares). Data points represent mean (± SD/SEM, where available).

The *in vitro* concentration–response curves were translated to *in vivo* dose–response curves using the PBK models for reverse dosimetry and QIVIVE. This resulted in the predicted dose–response curves presented in Figure 7 on the left *y*-axis. Figure 7 also presents, for comparison, the reported *in vivo* dose–response curves for the most sensitive endpoint as taken from Figure 6A on the right *y*-axis. The results thus obtained reveal an adequate match between the predicted and actual experimentally obtained dose–response curves, with the predicted ED_50_ values differing only 1- to 1.4-fold from the *in vivo* ED_50_ value (Table 2).

**Table 2.** Established ED_50_ Values for the Predicted *In Vivo* Dose–Response Data and *In Vivo* Data for Rat and Mouse via Parenteral Administration

	ED_50_ (µg/kg bw)	Endpoint	Literature
Injection			
Rat			
Predicted	1	MEA Spike	Kasteel and Westerink (2017)
	0.7	MEA Spike	Nicolas *et al.*, (2014)
*In vivo*	**0.7**	Von Frey	Marcil *et al.*, (2006)
	10.4	Nociceptive block	Kohane *et al.*, (1998)
Mice			
Predicted	2.1	Cytotoxicity inhibition	Hamasaki *et al.*, (1996)
	1.5	Cytotoxicity inhibition	Yamashoji and Isshiki (2001)
	2.4	Cytotoxicity inhibition	Yeo *et al.*, (1996)
*In vivo*	**0.84**	No. visceral contractions	Marcil *et al.*, (2006)
	12	Time to death	Finch *et al.*, (2018)

The literature reported ED_50_ values used for comparison to the predicted ED_50_ values are printed in bold.

**Figure 8.**
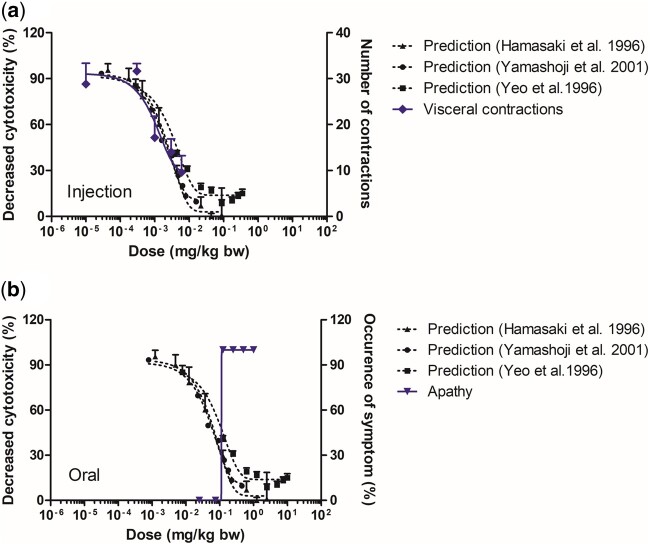
Predicted *in vivo* dose–response curves for tetrodotoxin in mice upon (A) injection (intramuscular model) and (B) oral administration (oral model). In blue the *in vivo* endpoints visceral contractions (diamonds; Marcil *et al.*, 2006; Figure 8A) and apathy (inverted triangles; Abal *et al.*, 2017; Figure 8B) displayed on right *y*-axes. The predictions were based on the mouse neuro-2a assay data reported by Hamasaki *et al.* (1996; triangles); Yamashoji and Isshiki (2001; circles), and Yeo *et al.* (1996; squares) displayed on the left *y*-axes. Data points represent mean (± SD/SEM, where available).

For mouse, both the IM model and the oral PBK model were used to translate the respective *in vitro* concentration–response data into *in vivo* dose–response data (Figure 8). For the parenteral route, the most sensitive endpoint was the number of visceral contractions for which the *in vivo* dose–response curve was provided by Marcil *et al.* (2006). Figure 8A presents a comparison of the *in vivo* experimental data (right *y*-axis) to the predicted dose–response curves (left *y*-axis) for TTX in mice. This comparison reveals that the predicted dose–response curves based on the *in vitro* data obtained in the neuro-2a assay are in line with the observed *in vivo* dose–response data as the predicted ED_50_ values vary 1.8-fold to a maximum of 3-fold from the *in vivo* ED_50_ value (Table 2). Apathy was the most sensitive endpoint for the oral route and therefore chosen for the comparison to the predicted dose–response curve upon oral administration of TTX (Figure 8B). Here too, the predicted dose–response curves (left *y*-axis) appear to be in accordance with the observed *in vivo* data (right *y*-axis) with the predicted ED_50_ values being at most up to 2.3-fold lower than the observed *in vivo* ED_50_ value (Table 3).

**Table 3.** Established ED_50_ Values for the Predicted *In Vivo* Dose–Response Data and *In Vivo* Data for Mouse via Oral Administration

	ED_50_ (µg/kg bw)	Endpoint	Literature
Oral			
Mice			
Predicted	61	Cytotoxicity inhibition	Hamasaki *et al.* (1996)
	42	Cytotoxicity inhibition	Yamashoji and Isshiki (2001)
	69	Cytotoxicity inhibition	Yeo *et al.* (1996)
*In vivo*	**96**	Apathy	Abal *et al.* (2017)
	560	Seizures/Numbness	
	223	Mortality	

The literature reported ED_50_ values used for comparison to the predicted ED_50_ values are printed in bold.

### Predicting TTX Neurotoxicity in Human and Estimating a Tentative PoD

**Figure 9.**
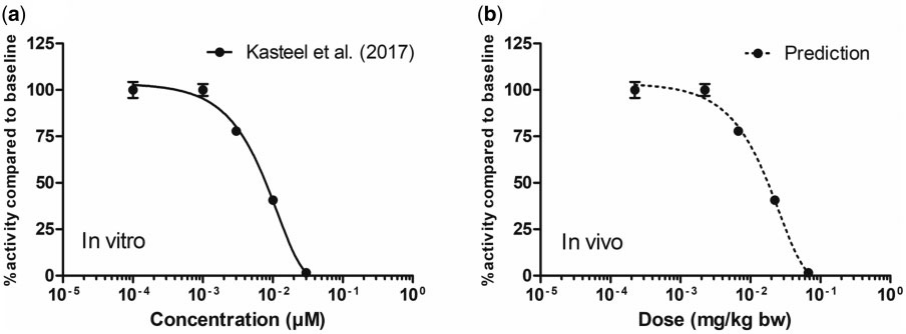
*In vitro* concentration–response curve of (A) tetrodotoxin in human-induced pluripotent stem cell (hIPSC)-derived iCell neurons in coculture with hIPSC-derived iCell astrocytes in the multielectrode array assay (Kasteel and Westerink 2017) and (B) the predicted *in vivo* dose–response curve acquired by physiologically based kinetic model facilitated reverse-dosimetry using a human oral model (B). Data points represent mean ± SEM.

Upon evaluation of the rat TTX model the model code was used to define a human oral PBK-model. For predicting TTX neurotoxicity in human by QIVIVE an *in vitro* data set reported by Kasteel and Westerink (2017) was used describing TTX toxicity towards human IPSC-derived iCell neurons in co-culture with hIPSC-derived iCell astrocytes exposed to TTX in the MEA (MEA assay; Figure 9A). The dose–response curve obtained of TTX in this MEA cell model was translated to an *in vivo* dose–response curve applying PBK model-based reverse-dosimetry with the human PBK-model resulting in an *in vivo* dose–response curve with an ED_50_ of 18 µg/kg bw (Figure 9B). Further BMD analysis on this predicted *in vivo* dose–response curve resulted in a BMD_10_ of 4.3 µg/kg bw, and a BMDL_10_ of 1.8 µg/kg bw (see Supplementary Figs. 3 and 4 and Supplementary Table 1 for details). Taking the BMDL_10_ as a PoD for the risk assessment of TTX and using a factor 10 for interindividual variability would result in an ARfD of 0.18 µg/kg bw. This tentative PoD is only 1.4-fold different from the previously established ARfD by EFSA of 0.25 µg/kg bw based on an acute toxicity study in mice (Abal *et al.*, 2017).

## DISCUSSION

TTX is an acute neurotoxin, which upon systemic exposure affects both action potential generation and impulse conduction by extracellular blockade of the voltage-gated sodium channels. The available ARfD of TTX (0.25 µg/kg bw), is derived from a study on mice in which TTX was dosed orally (via gavage; EFSA Panel on Contaminants in the Food Chain [CONTAM] *et al.*, 2017). Given the available data sets on acute toxicity of TTX in rodents and the many analogs of TTX for which experimental toxicity data are lacking, it is of interest to study whether the acute toxicity of TTX can be adequately predicted by a NAM based on *in vitro* and *in silico* data.

PBK modeling-based reverse dosimetry has proven to be a promising NAM to derive quantitative data, which can potentially be used in risk assessment to estimate *in vivo* toxicity in rodents and human (Chen *et al.*, 2018; Li *et al.*, 2017a; Louisse *et al.*, 2015; Ning *et al.*, 2019; Shi *et al.*, 2020; Strikwold *et al.*, 2017; Zhang *et al.*, 2018, 2020). This study aimed to assess the potential of using the PBK modeling-based reverse dosimetry approach as a NAM to predict the neurotoxicity of TTX in rodents, based on *in vitro* toxicity data obtained in the MEA assay using primary rat neonatal cortical cells or data obtained using the mouse neuro-2a assay.

Evaluation of the PBK model performance for TTX demonstrated its adequacy for predicting kinetic data for different routes of administration. In line with the results from Hong *et al.* (2017) who reported that < 10% of TTX was metabolized, the results of this study corroborated that hepatic metabolism does not contribute substantially to the systemic clearance of TTX (*in vitro* CL_int_: 1.6 × 10^−7^±0.01 ml/min/10^6^ cells; *in vivo* CL_int_: 1.15 × 10^−5^ l/h), while renal excretion plays a major role in TTX kinetics. Furthermore, the PBK modeling data of this study revealed that up to 86% of TTX clearance in the kidney could be ascribed to active transport by the proximal tubule cells. This active transport of TTX was previously also demonstrated in the renal proximal tubule cell line LLC-PK1 (Matsumoto *et al.*, 2017). In this *in vitro* study, TTX was shown to be primarily transported by the organic cation transporters and the organic cation/carnitine transporters. To a lesser extent organic anion transporters and multidrug resistance-associated proteins were involved, too. The PBK model evaluation of this study provides insight in the efficiency of this active transport and revealed that it contributes considerably to TTX clearance. To substantiate the values used in this article, it would be of interest to perform *in vitro* transport studies with TTX for the organic cation transporters in stably transfected cell lines such as the human embryonic kidney cell line HEK-293 and investigate to what extent such *in vitro* data can provide the kinetic data defined in this study by fitting the PBK model to available *in vivo* data for TTX kinetics.

The results obtained revealed that the NAM used in this study could adequately predict the dose–response curves for the selected most sensitive endpoints reported in the available *in vivo* studies. This, in spite of the fact that the spike rates used as readout in the MEA assay (Nicolas *et al.*, 2014) and the endpoint quantified in the MTT assay using the mouse neuroblastoma cell line, both used to generate the *in vitro* concentration–response curves, may detect TTX neurotoxicity based on different endpoints than the endpoints quantified in the *in vivo* neurotoxicity studies. This is possible because the underlying mode of action for all *in vivo* endpoints relates to the TTX mediated blocking of sodium channels. The reasons underlying the differential sensitivity of the various *in vivo* endpoints may relate to as yet unidentified differences in the toxicodynamics and/or toxicokinetics of TTX in the target tissue of interest underlying the respective adverse effects (mechanical allodynia, thermal nociceptive blocking, visceral contractions, apathy, and seizures).

Hence the question arises as to what extent the endpoints quantified in the MEA assay or the neuro-2a assay match these *in vivo* endpoints. Although the mechanism of action underlying all the *in vitro* and *in vivo* endpoints studied for TTX is blocking of the voltage-gated sodium channels, thereby interfering with the production of action potentials, it is of interest to consider the different endpoints in some more detail. For the rat *in vivo* data, the sensory neurons stimulated in the Von Frey hair test and the thermal nociceptive blocking test are nonvisceral (or somatic) sensory neurons that can respond to (noxious) events such as mechanical, (extreme) heat/cold, or chemical stimuli (Dubin and Patapoutian, 2010; Robinson and Gebhart, 2008). In both experiments, the hind paw of the rats was exposed to either mechanical stimuli by Von Frey filaments or heat stimuli by a hot plate (56°C) until paw withdrawal was observed. Apparently, enduring the pain of heat (uncomfortable sensation) requires higher doses of TTX than enduring the pinprick of a Von Frey filament until uncomfortable sensation, and the type of stimuli (mechanical, heat) and/or the underlying pathway determines how sodium channel blocking is perceived. Here, the Von Frey hair test seems to be the more sensitive endpoint than the thermal nociceptive blocking test. The underlying neuronal/neuromuscular processes to further explain this difference between the different *in vivo* endpoints lies beyond the scope of this study (Dubin and Patapoutian, 2010).

In the MEA assay, primary rat neonatal cortical neurons isolated from cortices form a network of inhibitory and excitatory cells with different subtypes and amongst them nonvisceral neurons (Masland, 2004; Nicolas *et al.*, 2014; Schnitzler *et al.*, 1999). In the MEA assay, the neuronal cells are directly exposed to TTX and show a decrease in activity compared with baseline with increasing concentrations of TTX. The sodium channel block is therefore directly measurable, whereas this effect *in vivo* is only indirectly noticeable via neuromuscular communication with the central nervous system. Nevertheless, the MEA assay provides a very sensitive endpoint; therefore, the *in vivo* endpoint chosen for the comparison to data predicted based on the *in vitro* assay should be as sensitive as possible.

A similar evaluation for *in vitro* endpoints and *in vivo* endpoints can be performed for the mouse assays. The *in vivo* data on mice, generated in the writhing test, are based on innervation of the visceral sensory neurons by exposure to acetic acid, which via the acid-sensing ion channels lead to pain sensation expressed as abdomen contraction together with twisting and turning of the trunk and arching of the back (Holzer, 2011; Marcil *et al.*, 2006; Robinson and Gebhart, 2008). These effects are decreased by increasing TTX concentrations blocking the sodium channels and preventing signal transduction. This endpoint appears much more sensitive than measuring the time of death that requires higher doses of TTX (Finch *et al.*, 2018). Comparing the endpoint of the TTX effect in the *in vivo* writhing test—decrease of visceral contractions—to the TTX effect in the *in vitro* neuro-2a assay—cell survival—suggests that these endpoints are not exactly the same in spite of the similar underlying mechanism of action. However, in spite of this apparent difference, the use of the neuro-2a assay for QIVIVE did provide adequate *in vivo* predictions for the writhing test. Similarly, outcomes of the *in vitro* embryonic stem cells test for developmental toxicity, detecting the inhibition of the development of mouse embryonic ES-D3 stem cells to beating cardiomyocytes, appeared to provide a suitable *in vitro* endpoint to predict a wide range of *in vivo* endpoints for developmental toxicity including malformations, number of live pups, and fetal body weight (Kamelia *et al.*, 2017; Li *et al.*, 2017b; Strikwold *et al.*, 2013).

With respect to neurotoxicity, previous studies already concluded that for determining the toxicity of neurotoxins *in vitro* the 2 most promising assays are the MEA assay (using rat primary neonatal cortical cells) and the mouse neuro-2a assay (Bodero *et al.*, 2018; Nicolas *et al.*, 2014, 2015). The results of this study reveal that these 2 assays are adequate to define concentration-dependent *in vitro* toxicity data for TTX for QIVIVE using PBK model-based reverse dosimetry. Moreover, using a human IPSC *in vitro* MEA assay showed to have potential to generate data for establishing a tentative PoD (BMDL_10_) for human TTX toxicity in line with the previously established ARfD by EFSA. To confirm this with more proof, more research should be conducted on the kinetics of TTX in human.

To recapitulate, in this study, we have successfully built a PBK model for the marine biotoxin TTX in rodents (rat, mouse) where renal excretion via active transport seems to play a major role in its kinetics. The results presented provide support for the use of this NAM for predicting the acute neurotoxicity of TTX (and its analogs). Thereby, a cautious attempt has been made to predict TTX toxicity in human using only *in vitro* and *in silico* data applying reverse-based dosimetry enabled by PBK-modeling and shows to have potential. 

## SUPPLEMENTARY DATA

Supplementary data are available at *Toxicological Sciences* online.

## FUNDING

This research was supported by BASF SE.

## DECLARATION OF CONFLICTING INTERESTS

The authors declared no potential conflicts of interest with respect to the research, authorship, and/or publication of this article.

## Supplementary Material

kfac022_Supplementary_Data
