# Supplementary material for: Use of Physiologically Based Kinetic Modeling-Facilitated Reverse Dosimetry to Predict In Vivo Acute Toxicity of Tetrodotoxin in Rodents
Source: Toxicol Sci. 2022 Feb 26;187(1):127–38. doi: 10.1093/toxsci/kfac022 (PMC9041554; doi:10.1093/toxsci/kfac022)
Supplement: kfac022_Supplementary_Data [file kfac022_supplementary_data.zip › toxsci-21-0523-File002.docx]

Supplementary material A for: “Use of physiologically based kinetic modeling-facilitated reverse dosimetry to predict in vivo acute toxicity of tetrodotoxin in rodents”

Model code for PBK-model built in Berkeley Madonna for rat

;Date: November 2019

;Species: Rats

;Compound: Tetrodotoxin (TTX)

;Compiled by: Mengying Zhang and Annelies Noorlander

;Organization: Wageningen University

;=====================================================================

;Physiological parameters

;=====================================================================

;tissue volumes >> reference: Brown et al.,1997 Table 21

BW = 0.240 ; body weight rat (from in vivo kinetic study, Hong et al. 2017)

VFc = 0.070 ; fraction of fat tissue

VLc = 0.034 ; fraction of liver tissue

VKc = 0.007 ; fraction of kidney tissue

VBc = 0.074 ; fraction of blood

VRc = 0.091 ; fraction of rapidly perfused tissue

VSc = 0.724 ; fraction of slowly perfused tissue

VF = VFc*BW ;(L or Kg) ; volume of fat tissue (calculated)

VL = VLc*BW ;(L or Kg) ; volume of liver tissue (calculated)

VK = VKc*BW ;(L or Kg) ; volume of kidney tissue (calculated)

VB = VBc*BW ;(L or Kg) ; volume of blood (calculated)

VR = VRc*BW ;(L or Kg) ; volume of richly perfused tissue (calculated)

VS = VSc*BW ;(L or Kg) ; volume of slowly perfused tissue (calculated)

;--------------------------------------------------------------------------------------------------------------------

;blood flow rates >> reference: Brown et al.,1997 Table 25

QC = 15*BW^0.74 ;cardiac output (L/hr) ;reference: Brown et al., 1997 p. 453

QFc = 0.070 ; fraction of blood flow to fat

QLc = 0.174 ; fraction of blood flow to liver

QKc = 0.141 ; fraction of blood flow to kidney

QRc = 0.093 ; fraction of blood flow to rapidly perfused tissue

QSc = 0.522 ; fraction of blood flow to slowly perfused tissue

QF = QFc*QC ;(L/hr) ; blood flow to fat tissue (calculated)

QL = QLc*QC ;(L/hr) ; blood flow to liver tissue (calculated)

QK = QKc*QC ;(L/hr) ; blood flow to kidney tissue (calculated)

QR = QRc*QC ;(L/hr) ; blood flow to rapidly perfused tissue (calculated)

QS = QSc*QC ;(L/hr) ; blood flow to slowly perfused tissue (calculated)

;=====================================================================

;Partition Coefficients

;=====================================================================

; Partition coefficients are derived from Rodgers and Rowland using the QIVIVE tool from A. Punt www.qivivetools.wur.nl

PF = 0.46 ;fat/blood partition coefficient

PL = 4.29 ;liver/blood partition coefficient

PK = 4.70 ;kidney/blood partition coefficient

PR = 4.29 ;rapidly perfused tissue/blood partition coefficient

PS = 0.95 ;slowly perfused tissue/blood partition coefficient

;=====================================================================

;Kinetic parameters

;=====================================================================

kb = 50 ; (/hr) rate from muscle to blood

ka = 0.18 ; (/hr) uptake rate from stomach

kn = 1000000 ; (/hr) rate needle to blood

;--------------------------------------------------------------------------------------------------------------------

;Metabolism liver

;metabolism of TTX, scaled maximum rate of metabolism

CLint = CellDL*VL*(CLintHep*60*1E-3) ;(L/hr) ;Hepatic clearance

CLintHep = 0.00000016 ;(ml/min/million cells) ;Hepatic clearance derived from hepatocytes

CellDL = 135*1000 ;(million cells/kg liver)

;Hepatocyte number in rat = 1.35 *10E8 cells/g liver = 135 million cells/g liver = 135*1000 million cells/ kg liver

;reference: (Houston et al., 1994)

;--------------------------------------------------------------------------------------------------------------------

;Excretion from kidney

;Active uptake of TTX is based on the OCT2 transporter

VmaxTTXc = 180 ;{pmol/min/mg protein}

VmaxTTX = (VmaxTTXc/1000000)*60*SF*VK*1000 ;{umol/hr}

;300 mg prot./g kidney (Kumar et al. 2018)

;only 70% of whole kidney is cortex --> in cortex tubule cells thus OCT-2s are present (Kumar et al. 2018)

; 300 * 0.7 = 210 mg prot./g kidney

Km = 2 ; {uM} transport constant of TTX

SF = 210 ; mg/g protein

;=====================================================================

;Run settings

;=====================================================================

;Molecular weight

MW = 319.27 ; Molecular weight TTX (PubChem)

;Intramuscular dose is 6 ug/kg bw = 0.006 mg/kg bw (Hong et al., 2017)

IMDOSEmg = 0.006 ; (mg/kg bw) ; IMDOSEmg = given IM dose in mg/kg bw

IMDOSEumol2 = IMDOSEmg*1E-3/MW*1E6 ;(umol/ kg bw)

;IMDOSEumol2 = given intramuscular dose recalculated to umol/kg bw

IMDOSEumol = IMDOSEumol2*BW ;IMDOSEumol = umol given IM

;Oral dose is 100 ug/kg bw = 0.1 mg/kg bw (Hong et al., 2018)

;Bioavailability via oral route = 6.7%

ODOSEmg = 0.1*0.067 ; (mg/kg bw) ; ODOSEmg = given oral dose in mg/kg bw

ODOSEumol2 = ODOSEmg*1E-3/MW*1E6 ;(umol/ kg bw)

;ODOSEumol2 = given oral dose recalculated to umol/kg bw

ODOSEumol=ODOSEumol2*BW ; ODOSEumol = umol given oral

;Intravenous dose is 6 ug/kg bw = 0.006 mg/kg bw (Hong et al., 2018)

IVDOSEmg = 0.006 ; (mg/kg bw) ; IVDOSEmg = given IV dose in mg/kg bw

IVDOSEumol2 = IVDOSEmg*1E-3/MW*1E6 ;(umol/ kg bw)

;IVDOSEumol2 = given intravenous dose recalculated to umol/kg bw

IVDOSEumol = IVDOSEumol2*BW ;IVDOSEumol = umol given IV

;time

Starttime = 0 ; in hr

Stoptime = 48 ; in hr

DTMIN = 1e-6 ; minimum integration time (DT)

DTMAX = 0.0015 ; maximum integration time (DT)

;=====================================================================

;Model calculations

;=====================================================================

; model of TTX

;--------------------------------------------------------------------------------------------------------------------

;ANe = amount in needle, umol

ANe' = -kn*ANe ;(umol/hr)

Init ANe = IVDOSEumol

;--------------------------------------------------------------------------------------------------------------------

;stomach compartment

;Ast = amount in stomach, umol

Ast' = -ka*Ast ;(umol/hr)

Init ASt = ODOSEumol

;--------------------------------------------------------------------------------------------------------------------

;liver compartment

;AL = Amount TTX in liver tissue, umol

AL' = ka*ASt + QL*(CB - CVL) - AMint' ;(umol/hr)

Init AL = 0

CL = AL/VL

CVL = CL/PL

;AMint = amount TTX metabolized

AMint' = CLint*CVL ;(umol/hr)

init AMint = 0

;--------------------------------------------------------------------------------------------------------------------

; kidney compartment

; AK = Amount of TTX in kidney tissue, umol

AK' = QK*(CB-CVK) -GF'-AKe' ;(umol/hr)

Init AK = 0

CK = AK/VK

CVK = CK/PK

;GFR = glomerular filtration rate {L/hr} ; Walton, et al 2004

;GFR rat = 5.2 ; mL/min/kg bw

GFR = 0.0052*BW*60 ;L/hr

;GF = glomerular filtration of TTX (umol/hr)

GF' = GFR*(CVK*Fub)

Init GF = 0

;Fub = fraction unbound of TTX

Fub = 1

;AKe = amount TTX actively excreted from the kidney (umol)

;AKe' = amount TTX actively excreted from the kidney in time (umol/hr)

AKe' = VmaxTTX*(CVK*Fub)/(Km + (CVK*Fub))

Init AKe = 0

;--------------------------------------------------------------------------------------------------------------------

;fat compartment

;AF = Amount TTX in fat tissue (umol)

AF' = QF*(CB-CVF) ;(umol/hr)

Init AF = 0

CF = AF/VF

CVF = CF/PF

;--------------------------------------------------------------------------------------------------------------------

;Intramuscular (IM) injection site compartment

;AInj = Amount TTX in IM injection site compartment (umol)

AInj' = -kb *AInj ;(umol/hr)

Init AInj = IMDOSEumol

;--------------------------------------------------------------------------------------------------------------------

;tissue compartment richly perfused tissue

;AR = Amount TTX in rapidly perfused tissue (umol)

AR' = QR*(CB-CVR) ;(umol/hr)

Init AR = 0

CR = AR/VR

CVR = CR/PR

;--------------------------------------------------------------------------------------------------------------------

;tissue compartment slowly perfused tissue

;AS = Amount TTX in slowly perfused tissue (umol)

AS' = QS*(CB-CVS) ;(umol/hr)

Init AS = 0

CS = AS/VS

CVS = CS/PS

;--------------------------------------------------------------------------------------------------------------------

; blood compartment

;AB = Amount TTX in blood (umol)

AB' = (QF*CVF + QL*CVL + QK*CVK + QS*CVS + QR*CVR + kb*AInj + kn*ANe - QC*CB) ;(umol/hr)

Init AB = 0

CB = AB/VB

;=====================================================================

;Mass balance calculations

;=====================================================================

Total = IMDOSEumol + ODOSEumol + IVDOSEumol

Calculated = ANe + ASt + AL + AMint + AF + AK + AKe + GF + AInj + AS + AR + AB

ERROR=((Total-Calculated)/Total+1E-30)*100

MASSBBAL=Total-Calculated + 1

Model code for PBK-model built in Berkeley Madonna for mouse

;Date: March 2021

;Species: Mouse

;Compound: Tetrodotoxin (TTX)

;Compiled by: Mengying Zhang and Annelies Noorlander

;Organization: Wageningen University

;=======================================

;Physiological parameters

;=====================================================================

;tissue volumes >> reference: Hall et al., 2012 Table 2

BW = 0.03 ; body weight mouse (kg)

VFc = 0.07 ; fraction of fat tissue

VLc = 0.055 ; fraction of liver tissue

VKc = 0.017 ; fraction of kidney tissue

VBc = 0.067 ; fraction of blood

VRc = 0.137 ; fraction of rapidly perfused tissue

VSc = 0.654 ; fraction of slowly perfused tissue

VF = VFc*BW ;(L or Kg) ; volume of fat tissue (calculated)

VL = VLc*BW ;(L or Kg) ; volume of liver tissue (calculated)

VK = VKc*BW ;(L or Kg) ; volume of kidney tissue (calculated)

VB = VBc*BW ;(L or Kg) ; volume of blood (calculated)

VR = VRc*BW ;(L or Kg) ; volume of richly perfused tissue (calculated)

VS = VSc*BW ;(L or Kg) ; volume of slowly perfused tissue (calculated)

;--------------------------------------------------------------------------------------------------------------------

;blood flow rates >> reference: Hall et al., 2012 Table 4

;Cardiag output (QC) is based on the formula for mice found in Brown et al., 1997: 0.257*BW^0.75 {L/min}

QC = 15.4*BW^0.75 ;(L/hr) ; cardiac output

QFc = 0.070 ; fraction of blood flow to fat

QLc = 0.158 ; fraction of blood flow to liver

QKc = 0.114 ; fraction of blood flow to kidney

QRc = 0.516 ; fraction of blood flow to rapidly perfused tissue

QSc = 0.142 ; fraction of blood flow to slowly perfused tissue

QF = QFc*QC ;(L/hr) ; blood flow to fat tissue (calculated)

QL = QLc*QC ;(L/hr) ; blood flow to liver tissue (calculated)

QK = QKc*QC ;(L/hr) ; blood flow to kidney tissue (calculated)

QR = QRc*QC ;(L/hr) ; blood flow to rapidly perfused tissue (calculated)

QS = QSc*QC ;(L/hr) ; blood flow to slowly perfused tissue (calculated)

;=====================================================================

;Partition Coefficients

;=====================================================================

; Partition coefficients are derived from Rodgers and Rowland using the QIVIVE tool from A. Punt www.qivivetools.wur.nl

PF = 0.46 ;fat/blood partition coefficient

PL = 4.29 ;liver/blood partition coefficient

PK = 4.70 ;kidney/blood partition coefficient

PR = 4.29 ;rapidly perfused tissue/blood partition coefficient

PS = 0.95 ;slowly perfused tissue/blood partition coefficient

;=====================================================================

;Kinetic parameters

;=====================================================================

kb = 50 ; (/hr) intramuscular uptake rate constant

ka = 0.18 ; (/hr) uptake rate from stomach

kn = 1000000 ; (/hr) uptake rate from needle

;--------------------------------------------------------------------------------------------------------------------

;Metabolism liver

;metabolism of TTX, scaled maximum rate of metabolism

CLint = CellDL*VL*(CLintHep*60*1E-6) ;(L/hr) ;Hepatic clearance

CLintHep = 0.00016 ;(ul/min/million cells) ;Hepatic clearance derived from hepatocytes

CellDL = 135*1000 ;(million cells/kg liver)

;Hepatocyte number in rat = 1.35 *10E8 cells/g liver = 135 million cells/g liver = 135*1000 million cells/ kg liver

;reference: (Sohlenius-Sternbeck., 2006)

;--------------------------------------------------------------------------------------------------------------------

;Excretion from kidney

;Active uptake of TTX is based on the OCT2 transporter

VmaxTTXc = 180 ;{pmol/min/mg protein}

VmaxTTX = (VmaxTTXc/1000000)*60*SF*VK*1000 ;{umol/hr}

;300 mg prot./g kidney (Kumar et al. 2018)

;only 70% of whole kidney is cortex --> in cortex tubule cells thus OCT-2s are present (Kumar et al. 2018)

; 300 * 0.7 = 210 mg prot./g kidney

Km = 2 ; {uM} transport constant of TTX

SF = 210 ; mg/g protein

;=====================================================================

;Run settings

;=====================================================================

;Molecular weight

MW = 319.27 ; Molecular weight TTX (PubChem)

;Intramuscular dose is 6 ug/kg bw = 0.006 mg/kg bw (Hong et al., 2017)

IMDOSEmg = 0.006 ; (mg/kg bw) ; IMDOSEmg = given IM dose in mg/kg bw

IMDOSEumol2 = IMDOSEmg*1E-3/MW*1E6 ;(umol/ kg bw)

;IMDOSEumol2 = given intramuscular dose recalculated to umol/kg bw

IMDOSEumol = IMDOSEumol2*BW ;IMDOSEumol = umol given IM

;Oral dose is 100 ug/kg bw = 0.1 mg/kg bw (Hong et al., 2018)

;Bioavailability via oral route = 6.7%

ODOSEmg = 0.1*0.067 ; (mg/kg bw) ; ODOSEmg = given oral dose in mg/kg bw

ODOSEumol2 = ODOSEmg*1E-3/MW*1E6 ;(umol/ kg bw)

;ODOSEumol2 = given oral dose recalculated to umol/kg bw

ODOSEumol=ODOSEumol2*BW ; ODOSEumol = umol given oral

;Intravenous dose is 6 ug/kg bw = 0.006 mg/kg bw (Hong et al., 2018)

IVDOSEmg = 0.006 ; (mg/kg bw) ; IVDOSEmg = given IV dose in mg/kg bw

IVDOSEumol2 = IVDOSEmg*1E-3/MW*1E6 ;(umol/ kg bw)

;IVDOSEumol2 = given intravenous dose recalculated to umol/kg bw

IVDOSEumol = IVDOSEumol2*BW ;IVDOSEumol = umol given IV

;=====================================================================

;Model calculations

;=====================================================================

; model of TTX

;--------------------------------------------------------------------------------------------------------------------

;ANe = amount in needle, umol

ANe' = -kn*ANe ;(umol/hr)

Init ANe = IVDOSEumol

;--------------------------------------------------------------------------------------------------------------------

;stomach compartment

;Ast = amount in stomach, umol

Ast' = -ka*Ast ;(umol/hr)

Init ASt = ODOSEumol

;-------------------------------------------------------------------------------------------------------------------

;liver compartment

;AL = Amount TTX in liver tissue, umol

AL' = ka*ASt + QL*(CB - CVL)-AMint' ;(umol/hr)

Init AL = 0

CL = AL/VL

CVL = CL/PL

;AMint = amount TTX metabolized

AMint' = CLint*CVL ;(umol/hr)

init AMint = 0

;--------------------------------------------------------------------------------------------------------------------

; kidney compartment

; AK = Amount of TTX in kidney tissue, umol

AK' = QK*(CB-CVK)-GF'-AKe' ;(umol/hr)

Init AK = 0

CK = AK/VK

CVK = CK/PK

;GFR = glomerular filtration of TTX rate (L/hr) ; reference (Walton et al. 2004)

;GFR in mouse is 14 mL/min/kg BW

GFR = 0.014*BW*60 ;{L/hr}

;GF = glomerular filtration of TTX (umol/hr)

GF'= GFR*(CVK*fub)

Init GF = 0

; fub = fraction unbound of TTX ; obtained from QSAR/RED exp

Fub = 1

;AKe = amount TTX actively excreted from the kidney (umol)

;AKe' = amount TTX actively excreted from the kidney in time (umol/hr)

AKe' = VmaxTTX*(CVK*Fub)/(Km + (CVK*Fub))

Init AKe = 0

;--------------------------------------------------------------------------------------------------------------------

;fat compartment

;AF = Amount TTX in fat tissue (umol)

AF' = QF*(CB-CVF) ;(umol/hr)

Init AF = 0

CF = AF/VF

CVF = CF/PF

;--------------------------------------------------------------------------------------------------------------------

;Intramuscular (IM) injection site compartment

;AInj = Amount TTX in IM injection site compartment (umol)

AInj' = -kb *AInj ;(umol/hr)

Init AInj = IMDOSEumol

;--------------------------------------------------------------------------------------------------------------------

;tissue compartment richly perfused tissue

;AR = Amount TTX in rapidly perfused tissue (umol)

AR' = QR*(CB-CVR) ;(umol/hr)

Init AR = 0

CR = AR/VR

CVR = CR/PR

;--------------------------------------------------------------------------------------------------------------------

;tissue compartment slowly perfused tissue

;AS = Amount TTX in slowly perfused tissue (umol)

AS' = QS*(CB-CVS) ;(umol/hr)

Init AS = 0

CS = AS/VS

CVS = CS/PS

;--------------------------------------------------------------------------------------------------------------------

; blood compartment

;AB = Amount TTX in blood (umol)

AB' = (QF*CVF + QL*CVL + QK*CVK + QS*CVS + QR*CVR + kb*AInj +kn*ANe - QC*CB) ;(umol/hr)

Init AB = 0

CB = AB/VB

AUC' = CB ;umol*hr/L

Init AUC = 0

;=====================================================================

;Mass balance calculations

;=====================================================================

Total = IMDOSEumol + ODOSEumol + IVDOSEumol

Calculated = ANe + ASt + AL + AMint + AF + AK + AKe + GF + AInj + AS + AR + AB

ERROR=((Total-Calculated)/Total+1E-30)*100

MASSBBAL=Total-Calculated + 1

Model code for PBK-model built in Berkeley Madonna for human

;Date: December 2021

;Species: Human

;Compound: Tetrodotoxin (TTX)

;Compiled by: Annelies Noorlander

;Organization: Wageningen University

;=======================================

;Physiological parameters

;=====================================================================

;tissue volumes >> reference: Brown et al., 1997 (Table 21)

BW = 70 ; body weight human (kg)

VFc = 0.214 ; fraction of fat tissue

VLc = 0.026 ; fraction of liver tissue

VKc = 0.004 ; fraction of kidney tissue

VBc = 0.079 ; fraction of blood

VRc = 0.064 ; fraction of rapidly perfused tissue

VSc = 0.613 ; fraction of slowly perfused tissue

VF = VFc*BW ;(L or Kg) ; volume of fat tissue (calculated)

VL = VLc*BW ;(L or Kg) ; volume of liver tissue (calculated)

VK = VKc*BW ;(L or Kg) ; volume of kidney tissue (calculated)

VB = VBc*BW ;(L or Kg) ; volume of blood (calculated)

VR = VRc*BW ;(L or Kg) ; volume of richly perfused tissue (calculated)

VS = VSc*BW ;(L or Kg) ; volume of slowly perfused tissue (calculated)

;--------------------------------------------------------------------------------------------------------------------

;blood flow rates >> reference: Brown et al.,1997 Table 27

;Cardiac output (QC) for human is found in Brown et al., 1997 page 453

QC = 15*BW^0.74 ;(L/hr) ; cardiac output

QFc = 0.052 ; fraction of blood flow to fat

QLc = 0.227 ; fraction of blood flow to liver

QKc = 0.175 ; fraction of blood flow to kidney

QRc = 0.195 ; fraction of blood flow to rapidly perfused tissue

QSc = 0.351 ; fraction of blood flow to slowly perfused tissue

QF = QFc*QC ;(L/hr) ; blood flow to fat tissue (calculated)

QL = QLc*QC ;(L/hr) ; blood flow to liver tissue (calculated)

QK = QKc*QC ;(L/hr) ; blood flow to kidney tissue (calculated)

QR = QRc*QC ;(L/hr) ; blood flow to rapidly perfused tissue (calculated)

QS = QSc*QC ;(L/hr) ; blood flow to slowly perfused tissue (calculated)

;--------------------------------------------------------------------------------------------------------------------

;=====================================================================

;Partition Coefficients

;=====================================================================

; Partition coefficients are derived from Rodgers and Rowland using the QIVIVE tool from A. Punt www.qivivetools.wur.nl

PF = 0.46 ;fat/blood partition coefficient

PL = 4.29 ;liver/blood partition coefficient

PK = 4.70 ;kidney/blood partition coefficient

PR = 4.29 ;rapidly perfused tissue/blood partition coefficient

PS = 0.95 ;slowly perfused tissue/blood partition coefficient

;=====================================================================

;Kinetic parameters

;=====================================================================

ka = 0.18 ; (/hr) uptake rate from stomach

;--------------------------------------------------------------------------------------------------------------------

;Metabolism liver

;metabolism of TTX, scaled maximum rate of metabolism

CLint = CellDL*VL*(CLintHep*60*1E-6) ;(L/hr) ;Hepatic clearance

CLintHep = 0.00016 ;(ul/min/million cells) ;Hepatic clearance derived from hepatocytes

CellDL = 139*1000 ;(million cells/kg liver)

;Hepatocyte number in human = 1.39 *10E8 cells/g liver = 139 million cells/g liver = 139*1000 million cells/ kg liver

;reference: (Sohlenius-Sternbeck., 2006)

;--------------------------------------------------------------------------------------------------------------------

;Excretion from kidney

;Active uptake of TTX is based on the OCT2 transporter

VmaxTTXc = 180 ;{pmol/min/mg protein}

VmaxTTX = (VmaxTTXc/1000000)*60*SF*VK*1000 ;{umol/hr}

;300 mg prot./g kidney (Kumar et al. 2018)

;only 70% of whole kidney is cortex --> in cortex tubule cells thus OCT-2s are present (Kumar et al. 2018)

; 300 * 0.7 = 210 mg prot./g kidney

Km = 2 ; {uM} transport constant of TTX

SF = 210 ; mg/g protein

;=====================================================================

;Run settings

;=====================================================================

;Molecular weight

MW = 319.27 ; Molecular weight TTX (PubChem)

;Oral dose is 100 ug/kg bw = 0.1 mg/kg bw (Hong et al. 2018)

;Bioavailability via oral route = 6.7%

ODOSEmg = 0.1*0.067 ; (mg/kg bw) ; ODOSEmg = given oral dose in mg/kg bw

ODOSEumol2 = ODOSEmg*1E-3/MW*1E6 ;(umol/ kg bw)

;ODOSEumol2 = given oral dose recalculated to umol/kg bw

ODOSEumol=ODOSEumol2*BW ; ODOSEumol = umol given oral

;=====================================================================

;Model calculations

;=====================================================================

; model of TTX

;--------------------------------------------------------------------------------------------------------------------

;stomach compartment

;Ast = amount in stomach, umol

Ast' = -ka*Ast ;(umol/hr)

Init ASt = ODOSEumol

;-------------------------------------------------------------------------------------------------------------------

;liver compartment

;AL = Amount TTX in liver tissue, umol

AL' = ka*ASt + QL*(CB - CVL)-AMint' ;(umol/hr)

Init AL = 0

CL = AL/VL

CVL = CL/PL

;AMint = amount TTX metabolized

AMint' = CLint*CVL ;(umol/hr)

init AMint = 0

;--------------------------------------------------------------------------------------------------------------------

; kidney compartment

; AK = Amount of TTX in kidney tissue, umol

AK' = QK*(CB-CVK)-GF'-AKe' ;(umol/hr)

Init AK = 0

CK = AK/VK

CVK = CK/PK

;GFR = glomerular filtration of TTX rate (L/hr) ; reference (Walton et al. 2004)

;GFR in human is 1.8 mL/min/kg BW

GFR = 0.0018*BW*60 ;{L/hr}

; GF = glomerular filtration of TTX (umol/hr)

GF'= GFR*(CVK*fub)

Init GF = 0

; fub = fraction unbound of TTX ; obtained from QSAR/RED exp

Fub = 1

;AKe = amount TTX actively excreted from the kidney (umol)

;AKe' = amount TTX actively excreted from the kidney in time (umol/hr)

AKe' = VmaxTTX*(CVK*Fub)/(Km + (CVK*Fub))

Init AKe = 0

;--------------------------------------------------------------------------------------------------------------------

;fat compartment

;AF = Amount TTX in fat tissue (umol)

AF' = QF*(CB-CVF) ;(umol/hr)

Init AF = 0

CF = AF/VF

CVF = CF/PF

;--------------------------------------------------------------------------------------------------------------------

;tissue compartment richly perfused tissue

;AR = Amount TTX in rapidly perfused tissue (umol)

AR' = QR*(CB-CVR) ;(umol/hr)

Init AR = 0

CR = AR/VR

CVR = CR/PR

;--------------------------------------------------------------------------------------------------------------------

;tissue compartment slowly perfused tissue

;AS = Amount TTX in slowly perfused tissue (umol)

AS' = QS*(CB-CVS) ;(umol/hr)

Init AS = 0

CS = AS/VS

CVS = CS/PS

;--------------------------------------------------------------------------------------------------------------------

; blood compartment

;AB = Amount TTX in blood (umol)

AB' = (QF*CVF + QL*CVL + QK*CVK + QS*CVS + QR*CVR +kn*ANe - QC*CB) ;(umol/hr)

Init AB = 0

CB = AB/VB

AUC' = CB ;umol*hr/L

Init AUC = 0

;=====================================================================

;Mass balance calculations

;=====================================================================

Total = IMDOSEumol + ODOSEumol + IVDOSEumol

Calculated = ANe + ASt + AL + AMint + AF + AK + AKe + GF + AS + AR + AB

ERROR=((Total-Calculated)/Total+1E-30)*100

MASSBBAL=Total-Calculated + 1
