# Supplementary material for: Use of Physiologically Based Kinetic Modeling-Facilitated Reverse Dosimetry to Predict In Vivo Acute Toxicity of Tetrodotoxin in Rodents
Source: Toxicol Sci. 2022 Feb 26;187(1):127–38. doi: 10.1093/toxsci/kfac022 (PMC9041554; doi:10.1093/toxsci/kfac022)
Supplement: kfac022_Supplementary_Data [file kfac022_supplementary_data.zip › toxsci-21-0523-File003.docx]

Supplementary material B for: “Use of physiologically based kinetic modeling-facilitated reverse dosimetry to predict in vivo acute toxicity of tetrodotoxin in rodents”

Figure S1. Normalized sensitivity coefficients of PBK model parameters in rats for the predicted Cmax of TTX in blood after (a) oral administration of 6 µg/kg bw and (b) intramuscular administration of 6 µg/kg bw. Model parameters with normalized sensitivity coefficients with an absolute value higher than 0.1 are shown. VKc = volume of the kidneys, QKc = fraction of blood flow to the kidneys, QSc = fraction of blood flow to the slowly perfused tissues, PS = partition coefficient of slowly perfused tissue, Vmax = maximum rate of TTX transport via OCT2, Km = Michaelis-Menten constant of TTX transport via OCT2, SF = scaling factor, ka = absorption rate constant, VBc = volume of the blood, kb = rate of uptake after intramuscular injection

Figure S2. Predicted concentration time curves of TTX in whole blood of rat (striped lines) dosed TTX via (a) oral (diamonds), (b) IM (squares) and (c) IV (circles) administration based on passive glomerular filtration. The literature data reported as plasma concentrations were adjusted to blood concentrations assuming a blood:plasma ratio of 0.42 (Hong et al. 2017). Dosage used: oral 100 µg/kg bw with 6.7% bioavailability, IM and IV 6 µg/kg bw with 100% bioavailability. Data points represent mean (± SD/SEM, where available).

Fitted models

Table S1. Results BMD modeling of predicted human dose-response data, applying model averaging

| **Model** | **converged** | **loglik** | **npar** | **AIC** |
| --- | --- | --- | --- | --- |
| full model | yes | 16.75 | 5 | -23.5 |
| null model | yes | -5.21 | 2 | 14.42 |
| Expon. m3- | yes | 15.43 | 4 | -22.86 |
| Expon. m5- | yes | 16.74 | 5 | -23.48 |
| Hill m3- | yes | 15.44 | 4 | -22.88 |
| Hill m5- | yes | 16.72 | 5 | -23.44 |
| Inv.Expon. m3- | yes | 16.03 | 4 | -24.06 |
| Inv.Expon. m5- | yes | 16.49 | 5 | -22.98 |
| LN m3- | yes | 15.77 | 4 | -23.54 |
| LN m5- | yes | 16.53 | 5 | -23.06 |
| **Weights for model averaging** | | | | |
| EXP | HILL | INVEXP | LOGN |  |
| 0.23 | 0.23 | 0.31 | 0.24 |  |
| **Final BMD values** | | | | |
| Endpoint | Subgroup | BMDL | BMDU |  |
| Response | all | 0.00178 | 0.0062 |  |

Figure S3. Bootstrap curves based on model averaging for the predicted human TTX dose-response data.

Figure S4. BMD modeling results of individual models for the predicted human TTX dose-response data. a) Exponential model 5, b) Hill model 5, c) Inverted exponential model 3, d) Log normal model 3
